# The ^13^C method as a robust alternative to ^14^C-based measurements of primary productivity in the Mediterranean Sea

**DOI:** 10.1093/plankt/fby031

**Published:** 2018-08-11

**Authors:** Daffne C López-Sandoval, Antonio Delgado-Huertas, Susana Agustí

**Affiliations:** 1 King Abdullah University of Science and Technology (KAUST), Red Sea Research Center, Thuwal, Saudi Arabia; 2 Instituto Andaluz de Ciencias de la Tierra, Laboratorio De Biogeoquímica De Isótopos Estables, CSIC-UGR, Armilla, Spain

**Keywords:** primary productivity, ^13^C, ^14^C, exudation, Mediterranean Sea

## Abstract

Due to the increasing constraints on using the radioactive isotope ^14^C to measure primary productivity (^14^C-PP), we determined the surface carbon fixation rates in the Mediterranean Sea, using the alternative stable isotope ^13^C method (^13^C-PP). Rates obtained (^13^C-POCp) were compared with simultaneous ^14^C-POCp measurements in samples of different volumes (72 mL and 1.2 L). We also tested the variation of the percentage of dissolved primary production (PER), to the total productivity using organic and inorganic filters (^14^C method). ^13^C-POCp rates ranged from 0.4, in the Ionian basin, to 1.5 mgC m^−3^ h^−1^ in the Ligurian region. These results agreed with those found with the ^14^C-PP in 1.2-L samples (two sample *t*-test, *t* = 1.035, *df* = 22, *P* = 0.31). However, we found that ^14^C-POCp rates derived from 72-mL incubations were 46% lower than those measured with ^13^C-PP. The discrepancy between large and small volume incubations was likely due to differences in the number of large phytoplankton cells within the community. PER values measured with silver membrane filters yielded similar results than those obtained using polycarbonate filters. Our findings showed that when the sample size is carefully chosen, the ^13^C-PP provide comparable results to ^14^C-PP even in waters of low productivity in the Mediterranean Sea.

## INTRODUCTION

Assimilation of inorganic carbon via photosynthesis by phytoplankton represents the primary source of organic matter used to sustain the growth and metabolic demands of a large number of organisms in the oceans. The magnitude of this assimilation rate (primary productivity) constrains the biological flow of energy in the entire ecosystem and defines the overall biomass and productivity of a given area (Riley, 1944; Falkowski and Raven, 2007). Over the past decade, an increasing number of studies have predicted an expansion of oligotrophic regions of low productivity (Polovina *et al.*, 2008). This potential shift in productivity has profound implications for the ocean’s carbon balance. Therefore, it is crucial to accurately quantify phytoplankton carbon fixation rates to predict and understand the consequences of the changing marine ecosystems.

In oligotrophic systems, complex trophic relationships take place between small phytoplankton organisms (mostly < 2 μm), heterotrophic bacteria and small predators. These interactions result in the remineralization of the bulk of the produced organic matter that is made available (Sorokin, 1971; Azam *et al.*, 1983; Sherr and Sherr, 1994). Hence, the carbon export towards the interior of the ocean is mostly limited to dissolved organic matter that accumulates and sinks (Copin-Montégut and Avril, 1993; Carlson *et al.*, 1994).

The Mediterranean Sea is an oligotrophic basin in which seasonal productivity regimes have been extensively studied. The productivity cycle changes from higher carbon fixation rates over winter-early spring to lower fixation rates over the summer, due to a strong stratification of the water column (Estrada, 1996; Bosc *et al.*, 2004). During the summer months, carbon fixation rates can be as low as 95–190 mgC m^−2^ d^−1^ (Moutin and Raimbault, 2002; González *et al.*, 2008; López-Sandoval *et al.*, 2011). These low production rates are also accompanied by phytoplankton exudation rates of organic carbon that reach up to 30–40% (López-Sandoval *et al.*, 2011). Mesoscale and sub-mesoscale features in the Mediterranean Sea also play a significant role in the variability of carbon fixation rates (Estrada, 1996; Pinardi and Masetti, 2000). The influence of these hydrographic features is particularly relevant in the Western basin where primary production rates PP can reach values up to 1 g C m^−2^ d^−1^ in specific regions, throughout the year (Estrada, 1996; Moran and Estrada, 2001; Garcia-Gorriz and Carr, 2001; Estrada *et al.*, 2014). The dynamic nature and the well-studied productivity regime of the Mediterranean Sea provide an ideal setting for testing different methodological approximations for measuring the phytoplankton productivity.

Since Steeman Nielsen introduced the ^14^C method (Nielsen, 1952) to measure carbon fixation rates (^14^C-PP), this method has been extensively used, due to its high sensitivity and simplicity. However, in recent years, the use of ^14^C has become more restricted and sometimes even prohibited, due to concerns about handling radioactive material. One potential alternative consists of using the stable isotope ^13^C, instead of the radioactive one. The use of the ^13^C to measure PPP (^13^C-PP) was described by Slawyk *et al.* (1977). The method follows the same principle as the ^14^C-PP method: it involves tracking changes of the ratio ^13^C:^12^C relative to the total inorganic carbon pool (TCO_2_), using an enriched sample, generally NaH^13^CO_3_ (Cullen, 2001). The samples are later analysed by mass spectrometry to obtain the specific uptake rates. To convert the specific rates to absolute rates (mass volume^−1^ time^−1^), we must measure the carbon content in the sample. This analysis can be performed either simultaneously (Sakamoto *et al.*, 1984) or by filtering separate samples, later analysed on a CNH analyser (Sakamoto *et al.*, 1984; Slawyk *et al.*, 1984).

Results obtained from the ^13^C-PP and ^14^C-PP methods are generally in good agreement (Sakamoto *et al.*, 1984; Slawyk *et al.*, 1984; Hama *et al.*, 1983, 1993; Mousseau *et al.*, 1995; Regaudie-De-Gioux *et al.*, 2014). However, the magnitude of the results obtained by both protocols can display significant differences (Mousseau *et al.*, 1995; Regaudie-De-Gioux *et al.*, 2014), particularly in nutrient-depleted areas (Slawyk *et al.*, 1984). There are several reasons for variations between the two protocols to occur. For example, the initial conditions of the enclosed plankton assemblages can vary because the incubation volumes for ^13^C-PP tend to be larger than for ^14^C-PP (Sakamoto *et al.*, 1984; Slawyk *et al.*, 1984). The selection of the filter used in primary production experiments usually varies among the ^13^C-PP and ^14^C-PP protocols. The filter choice can determine the amount of organic carbon measured, as the retention capacity of the filter varies depending on the type of filter material (Lean and Burnison, 1979). Another potential source of discrepancy, found in earlier studies, is the incomplete combustion of the ^13^C-PP samples during the mass spectrometric analysis (Sakamoto *et al.*, 1984; Slawyk *et al.*, 1984; Mousseau *et al.*, 1995). However, over past decades, the use of elemental analysers in-line with isotope ratio mass spectrometers (EA-IRMS) has significantly improved the measurement process, and deficient combustions can hardly occur (Goodman and Brenna, 1992).

Some of these potential sources of discrepancies also apply to the ^14^C-PP protocol. Therefore, in our study, we aim to determine the variability of carbon fixation rates in the Mediterranean Sea during the summer period, using both carbon uptake protocols (^13^C-PP and ^14^C-PP). A comparison of the results obtained for both ^13^C-PP and ^14^C-PP permits assessment of the consistency of the data obtained for carbon fixation rates, and to help to determine the sensitivity of the ^13^C-PP protocol for measuring primary productivity in oligotrophic systems.

## METHODS

### Sampling and environmental variables

**Fig. 1.**
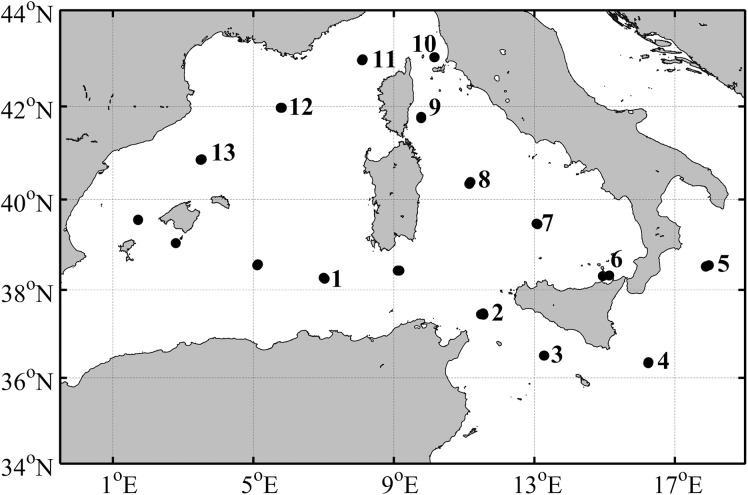
Cruise track for the ARGON cruise. The number indicates the position of the primary production stations.

Samples were collected between the 7 and 24 June 2016, during the Argon cruise on board of the R/V *L’ Garcia del Cid*, in the Mediterranean Sea, within a region delimited by coordinates 36–43°N and 3–17.5°E (Fig. 1). Vertical profiles of temperature, salinity and *in vivo* fluorescence were obtained from all casts, with a CTD SBE 911plus equipped with additional sensors that measure turbidity and oxygen concentration. Using the CTD data, we determined the depth of the pycnocline and calculated the Brunt–Väisalä frequency squared (*N*^2^) as a measurement of water column stratification. *N*^2^ is defined as *(−g/ρ*_*r*_) *dρ/dz*, where *g* is the acceleration due to gravity, *ρ* is the density and *z* is the depth (Gill, 1982).

Water samples from selected depths were collected between 7:00 and 9:00 am local time, using a rosette equipped with Niskin bottles. Samples obtained were used to determine the chlorophyll-a (Chl-*a*) concentration in the upper 200 m of the water column and the carbon fixation rates at the sea surface. For the Chl-*a* analysis, 200 mL samples were taken at six discrete depths (between 5 and 200 m) and filtered through Whatman GF/F filters (25 mm diameter). The filters were kept frozen at −20°C until further analysis back on land. Pigments were extracted using 90% acetone for 24 h and left in the dark at 4°C for 24 h. Chl-*a* concentration was estimated with the non-acidification technique using a Turner Design Trilogy Fluorometer, previously calibrated with pure Chl-*a*.

### Primary production measurements

We performed 12 long (9 h) simulated *in situ* (SIS) incubations to determine primary production in the particulate fraction (POCp), using the ^13^C-PP and ^14^C-PP methods (^13^C-POCp and ^14^C-POCp). We also performed 11 short (2–3 h) SIS incubations to determine dissolved and particulate primary production, using the ^14^C-PP with four different kinds of filters. For each incubation, surface water (5 m) was collected directly from the Niskin bottles and transferred to a 5-L acid-washed container for ^13^C-PP incubations, whereas samples for ^14^C-PP incubations were directly collected in polycarbonate bottles (PC) and polystyrene flasks. SIS incubations were performed in an on-deck incubator covered with neutral density mesh and maintained with recirculating surface water.

### ^13^C-POCp measurements

Water from the Niskin bottles was transferred into a 5-L carboy and enriched with 20 mL of ^13^C-labelled sodium bicarbonate solution (1.09 g of Na H^13^CO_3_, 99.8% ^13^C, in 1 L of Milli-Q water). The container was carefully shaken to homogenize the sample. The water was used to fill three light and one dark acid-washed 1-L (1.2-L final volume) PC bottles. In each bottle, the final concentration of ^13^C was 51.4 μmol ^13^C L^−1^. At the end of the incubation period (9 h), the entire bottle content was filtered onto a pre-combusted 25 mm Whatman GF/F filter. After filtration, the filters were exposed overnight to concentrated fumes of HCl to remove any inorganic carbon and subsequently kept frozen until analysis. Analyses were performed on desiccated filters, for 24 h, in a desiccator. To measure the isotopic enrichment (δ^13^C) and carbon content (POC, mgC m^−3^), a fraction of the GF/F filters (approximately one-third) was wrapped in tin capsules and subsequently measured on a Carlo Elba NC1500 (Milan, Italy) elemental analyser attached to a Delta Plus XL (ThermoQuest, Bremen, Germany) mass spectrometer (EA-IRMS).

The absolute amount of ^13^C incorporated into the particulate fraction (^13^C-POCp, mgC m^−3^ h^−1^), after the incubation time (*t*), was calculated as in equation (1):

(1) $$\begin{array}{c} {\;}^{13}C\text{-}\mathrm{POCp}\\ =([(\delta {\;}^{13}C_{\mathrm{light}}{-\delta }^{13}C_{\mathrm{dark}}{)/(\delta }^{13}C_{\mathrm{DIC}\;\mathrm{labelled}}{-\delta }^{13}C_{\mathrm{DIC}\;\mathrm{natural}})]\\ \times \mathrm{POC})/t \end{array}$$

where δ^13^C_light_ refers to the δ^13^C value obtained from filtration of the light bottles at the end of the incubation and δ^13^C_dark_ corresponds to the δ^13^C value obtained for the dark bottle (which in essence represents the natural abundance of ^13^C in the organic matter). δ^13^C _DIC labelled_ refers to the mix of natural dissolved inorganic carbon (^13^C-DIC) and ^13^C-DIC (from NaH^13^CO_3_) that was added to enrich the samples. δ^13^C-DIC labelled was calculated by taking into consideration the molar fraction and the atom percent content of ^13^C of the NaH^13^CO_3_ stock and the molar fraction and atom percent of ^13^C from the natural DIC pool. To determine the δ^13^C_DIC natural_ at each station, we took an aliquot of the sample before enrichment with NaH^13^CO_3_ and injected it into 12-mL vials pre-filled with helium and with five drops of 65% phosphoric acid. We then placed the vial in a vortex for 30 s. The vials remained at room temperature for equilibration, between 15 and 36 h (Salata *et al.*, 2000). The CO_2_ was separated from other residual gases by chromatography (with helium as a carrier gas), using a Gas Bench (Thermo Finnigan, Bremen, Germany) system interface and a mass spectrometer.

### ^14^C-POCp measurements

After collection, water was distributed in three sets of samples: one set of 1-L PC bottles (1.2 L final volume) and two sets of 70-mL polystyrene acid-washed flasks (72 mL final volume). Each set contained three light and one dark bottle. After we spiked the 1.2 L samples with 100 μCi of NaH^14^CO_3_ and the 72 mL ones with 20 μCi, all samples were immediately placed in an on-deck incubator. ^14^C-POCp samples were processed after 2–3 h incubations (from one of the two 72-mL samples set) and after 9 h in the other two samples sets (1.2-L samples set, and the remaining 72-mL sample set). The content of all the bottles was entirely filtered (<100 mm Hg) through 0.2-μm PC filters (25 mm in diameter, ∅). The inorganic ^14^C present in the filters was removed by exposing the filters to concentrated HCl fumes for 12 h and then placed in scintillation vials (4 mL) to which 3.4 mL of a scintillation cocktail was added. Radioactivity measurements of each sample were determined in a Beckman LS 6500 scintillation counter. The dark bottle DPM (disintegrations per minute) were subtracted from the light bottle DPM to calculate the production rates. We used a value of 25 700 mgC m^−3^ for the concentration of dissolved inorganic carbon and a value of 1.05 for the isotopic discrimination factor.

### ^14^C-DOCp measurements

Dissolved primary production (^14^C-DOCp) rates were determined after a 2–3 h incubation period, from the 72-mL samples sets, using four different types of filters, and after a 9 h incubation, using 0.2-μm PC filters (Whatman, 25 mm ∅). The different types of filters used during the short incubations were borosilicate (25 mm Whatman GF/F, 0.7 μm), silver membrane (SM) (25 mm Steriltech, 0.2 μm), polycarbonate (25 mm Whatman nucleopore, 0.2 μm) and mixed acetate and nitrate cellulose (25 mm Millipore GSWP, 0.22 μm). For each incubation, two 5-mL replicates were taken from each 72-mL bottle and were filtered under low-vacuum pressure. Filtrates were subsequently acidified to a pH of ~2 with 100 μL of 50% HCl and kept overnight in open scintillation vials (20 mL) placed on an orbital shaker. After the removal of inorganic ^14^C, 15 mL of scintillation cocktail was added to each filtrate. The radioactivity on each filtrate and filter was determined following the procedure previously detailed in ^14^C-POCp measurements section.

## RESULTS

### Environmental conditions and phytoplankton productivity in the western Mediterranean Sea

**Fig. 2.**
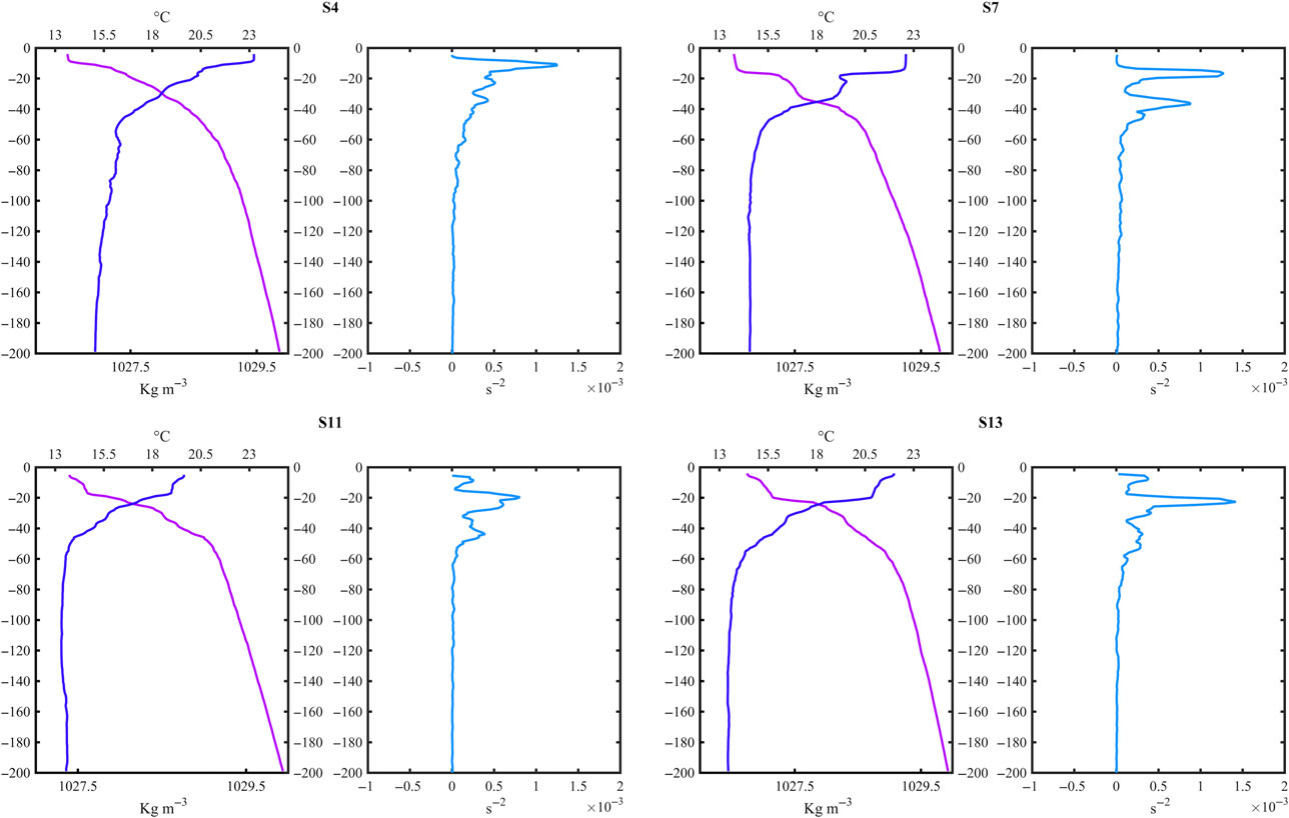
Characteristic vertical profiles of temperature (°C), density (kg m^−3^) and the square of the Brunt–Väisälä frequency (s^−2^) measured at different regions sampled during the cruise. S4—Ionian Sea, S7—Tyrrhenian Sea, S11—the Ligurian Sea and S13—the Balearic Sea. The number of the station is indicated in bold.

**Fig. 3.**
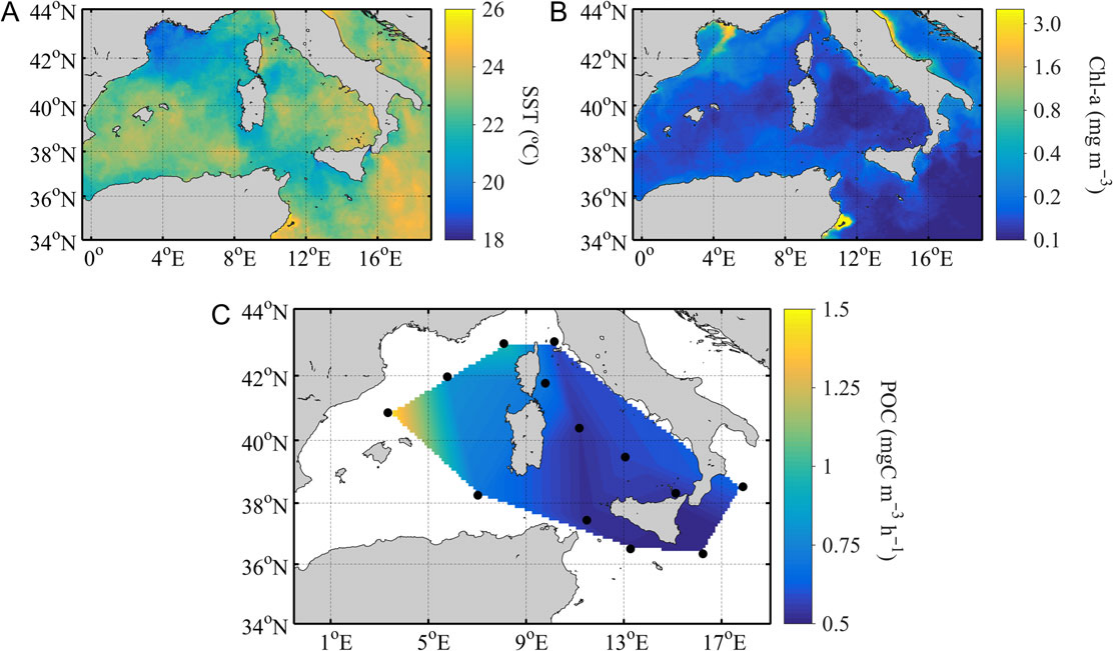
Satellite-derived, 32 days (1 June 2016 to 2 July 2016) composite maps of (**A**) sea surface temperature (Level-3, Global, 9 km) and (**B**) chlorophyll-a concentration (Level-3, Global, 4 km, of Chlorophyll a, OCI Algorithm). Both data sets were obtained from the Visible and Infrared Imager/Radiometer Suite (VIIRS), as processed by NASA GSFC Ocean Biology Processing Group (OBPG). (**C**) corresponds to the surface PP rates measured in the particulate fraction with the ^13^C-PP method (^13^C-POCp) (mgC m^−3^ h^−1^) during the cruise.

CTD data revealed that, in most of the stations, the water column structure was characterized by a sub-surface layer of warm and less dense waters constrained by a steep thermocline located around 15 and 20 m, and a second deeper layer between 40 and 50 m (Fig. 2). The boundary of the sub-surface layer agreed with the maximum value of the Brunt–Väisäla frequency (*N*^2^), which in some stations, also showed a second maximum between 40 and 50 m (Fig. 2). Sea surface temperature (SST) varied from 19 to 23°C and decreased sharply to ~15–16°C, after 40 m (Table I, Fig. 2 and 3A). Surface salinity ranged between 37.1 and 38.8, but at station 1, located in the Algerian Basin, the salinity was 36.8 (Table I), which is likely to be associated with the Modified Atlantic Water (MAW) (Millot, 1999). The phytoplankton Chl-*a* surface concentration remained below 0.15 mg m^−3^ (Fig. 3B), and the water column-integrated Chl-*a* ranged from 21 to 98 mg m^−2^ (Table I). Carbon fixation rates (the particulate fraction, ^13^C-POCp) ranged from 0.4 to 1.5 mgC m^−3^ h^−1^ (Table II, Fig. 3C), while carbon assimilation rates per unit Chl-a averaged 8 ± 2.3 mg^13^C (Chl*-a*)^−1^ h^−1^(Table II). The contribution of the dissolved primary production to the total productivity varied between 14 and 30% (mean = 21% ± 4 SD) (Table II).

**Table I:** Physical and biological properties measured during ARGON cruise in the Mediterranean Sea

Provinces	Station	Latitude	Longitude	SST	Salinity	Chl-a max depth	∫ Chl-*a*
		˚N	˚E	˚C	PSU	m	mg m^−2^
Algerian Basin	1	38.25	7.03	21.4	36.8	63	34
Sicily Strait	2	37.45	11.48	20.9	37.1	100	21
	3	36.51	13.27	21.6	37.3	77	49
North Ionian Sea	4	36.34	16.24	23.2	38.5	75	43
	5	38.53	17.88	21.9	38.8	80	44
Tyrrhenian Sea	6	38.32	15.13	22.6	37.9	90	56
	7	39.47	13.06	22.6	38.3	100	39
	8	40.38	11.18	21.7	38.2	90	53
	9	41.77	9.79	20.8	38.3	70	50
	10	43.03	10.15	20.3	38.1	95	61
Ligurian Sea	11	42.97	8.09	19.6	38.3	50	98
Algero-provençal Basin	12	41.97	5.78	19.1	38.2	50	85
Balearic Sea	13	40.86	3.34	22.0	38.3	60	71

SST is the sea surface temperature. Chl-*a* max depth, is the depth (m) where we found the maximum concentration of chlorophyll-a.∫ Chl-*a* corresponds to the water column-integrated chlorophyll-a concentration (mg m^−2^).

**Table II:** Means (±standard error) of PERs measured after 9-h incubation

Station	^13^C-POCp 1.2-L		^14^C-POCp 1.2-L		^14^C-POCp 72-mL		PER 72-mL		Assimilation numbers ^13^C-POCp (mg Chl*-a*)^−1^ h^−1^
	mgC m^−3^ h^−1^		mgC m^−3^ h^−1^		mgC m^−3^ h^−1^		%		
	Mean	Error	Mean	Error	Mean	Error	Mean	Error	
1	0.70	0.03	0.64	0.02	0.48	0.08	18	3.6	6.4
2	0.52	0.03	0.53	0.00	0.37	0.02	22	2.8	8.7
3	0.51	0.05	0.40	0.01	0.24	0.01	19	5.4	6.4
4	0.43	0.01	0.38	0.01	0.26	0.01	30	2.2	ND
5	0.63	0.02	0.52	0.00	0.30	0.04	24	2.5	12.6
6	0.54	0.02	0.53	0.02	0.32	0.02	18	4.5	4.2
7	0.58	0.04	0.42	0.01	0.26	0.01	24	2.6	8.3
8	0.53	0.03	0.41	0.00	0.23	0.02	27	5.2	8.8
9	0.70	0.05	0.73	0.01	0.39	0.04	19	1.3	7.8
10	0.58	0.06	0.61	0.00	0.35	0.02	21	2.0	5.8
11	0.97	0.06	0.86	0.01	0.50	0.01	18	1.0	6.9
12	0.80	0.12	0.70	0.03	0.38	0.01	17	1.2	5.7
13	1.47	0.07	ND	ND	0.68	0.07	14	2.0	14.7

^13^C-POCp are the PP rates in the particulate fraction measured in 1.2-L samples with the ^13^C-PP method. ^14^C-POCp are the particulate PP rates measured with the ^14^C-PP method in 1.2-L and 72-mL samples. PER corresponds to the percentage of dissolved primary production (DOCp) relative to total production rates (DOCp + POCp) from 72-mL ^14^C-PP incubations. Assimilation numbers are the ^13^C-POCp rates normalized by chlorophyll unit. ND, no data available.

We found that ^13^C-POCp rates between the Ligurian and the Balearic Sea were above the cruise average value (0.7 mg C m^−3^ h^−1^ ± 0.3) (Table II, stations 11–13). The high productivity in this region matched with the highest column-integrated phytoplankton biomass (above 70 mg m^−2^) (Table I) and with the lower PER values (14–18%) estimated during the cruise (Table II). Carbon fixation rates of 0.7 mgC m^−3^ h^−1^ were also found at station 9 (North of the Tyrrhenian Sea) and at station 1 (Algerian Basin) (Table II, Fig. 3C). The 32-day composite images of SST and Chl-*a* (Fig. 3A, B) suggest the presence of mesoscale structures in the vicinity of those stations; these oceanographic features can potentially enrich surface waters by bringing nutrients from deeper layers, thus promoting phytoplankton growth.

As we moved eastwards, through the Sicily strait towards the Ionian Sea, SST and salinity increased while phytoplankton carbon fixation rates decreased to values below the cruise average (Table II, Fig. 3C). In the stations located within this region, surface Chl-*a* concentration was the lowest, water column phytoplankton biomass (seen as Chl-*a* concentration) averaged 40 mg m^−2^ (SD, 13), and carbon fixation rates ranged from 0.43 to 0.63 mgC m^−3^ h^−1^ (Table II, Fig. 3C). We also observed PER values above the average percent (21%) in some of the stations, peaking in the Ionian basin (Table II); this highest PER (30%) value coincides with the lowest POCp rates measured in the region (Table II).

### Differences in PP rates between carbon fixation protocols

**Fig. 4.**
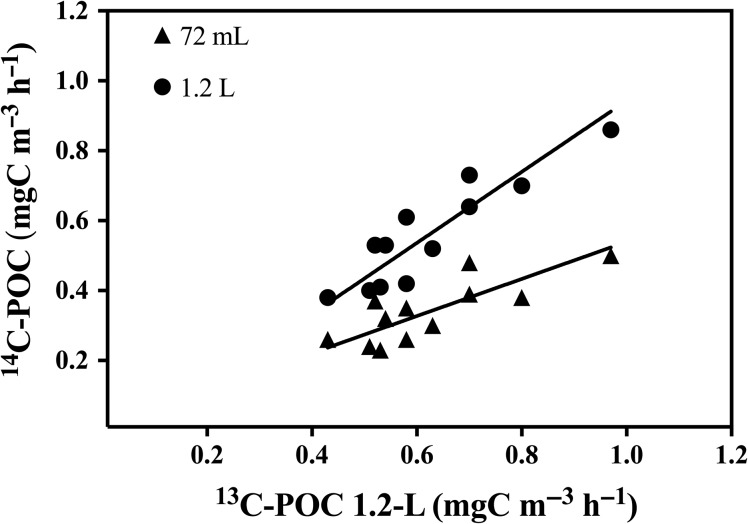
Relationship between particulate organic carbon production rates (POCp) obtained with the ^13^C-PP and ^14^C-PP method. Dark circles correspond to 1.2-L ^14^C-PP incubations and dark triangles correspond to 72-mL ^14^C-PP incubations. Incubation time was 9 h. The linear fit (reduced major axis regression) was ^14^C-POCp _72-mL_ = 0.53 (±0.1) *^13^C-POCp + 0.01 (±0.01) (*R*^2^ = 0.63, *n* = 12, *P* < 0.001) and ^14^C-POCp _1.2-L_ = 1.01 (±0.15) *^13^C-POCp + 0.1 (±0.01) (*R*^2^ = 0.82, *n* = 12, *P* < 0.001).

**Fig. 5.**
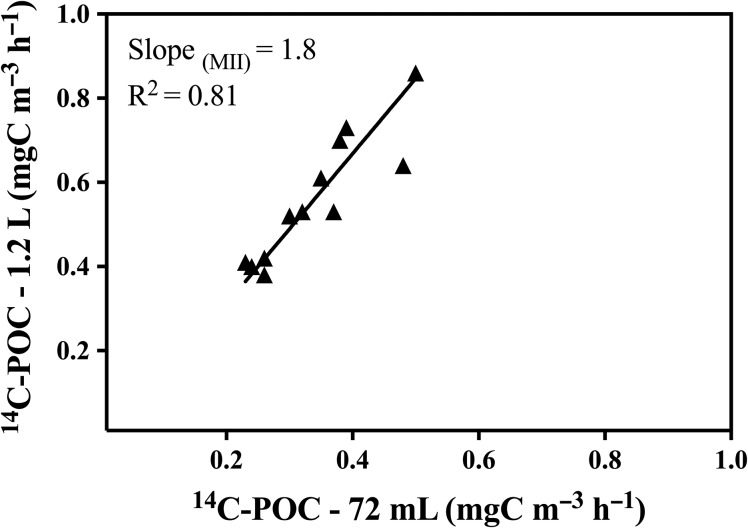
Relationship between 1.2-L and 72-mL particulate primary production (POCp) samples obtained with the ^14^C-PP method. Incubation time was 9 h.

In our study, ^13^C-POCp and ^14^C-POCp correlate significantly (*r* = 0.91, *P* < 0.001), and the observed degree of variability between ^13^C-POCp and ^14^C-POCp results were directly related to differences in sample volume (Fig. 4). ^13^C-POCp data were not significantly different from the ^14^C-POCp data obtained using 1.2-L samples (two sample *t*-test, *t* = 1.035, *df* = 22, *P* = 0.31), but differed significantly with ^14^C-PP data from 72-mL samples (two sample *t*-test, *t* = 5.68, *df* = 22, *P* = <0.001) (Table II, Fig. 4). Also the ^14^C-POCp rates from 1.2-L samples were significantly higher (two sample *t*-test, *t* = 4.36, *df* = 22, *P* = < 0.001) (~61%, on average) than those obtained from 72-mL ^14^C-POCp samples (Fig. 5).

Other variations due to the differences between the ^13^C-PP and ^14^C-PP protocols may have potentially biased carbon fixation results. Therefore, we analysed the effects of different filter membranes and the effect of incubation time on the POCp rates. ^14^C-POCp rates and PER values obtained from samples filtered through inorganic filters (borosilicate and SM) and through organic filters (polycarbonate and nitrocellulose) showed that ^14^C-POCp and PER results varied significantly depending on the type of filter used (Kruskal–Wallis, *H* = 9.55 and 14.96, *P* = 0.02 and <0.01, respectively). However, based on the ^13^C-POCp and ^14^C-POCp 1.2-L comparison (each one measured with a different type of filter; borosilicate and polycarbonate, respectively) (Fig. 4), our results suggest that the potential effect of the filter selection becomes negligible on samples greater than 100 mL.

**Fig. 6.**
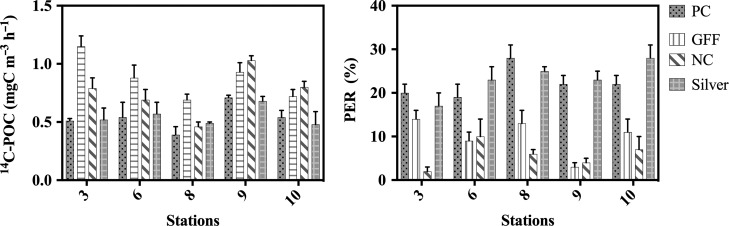
Particulate primary production (POCp) and the percentage of dissolved primary production (PER) related to total productivity (POCp + DOCp) obtained from 72-mL samples filtered through four different types of filter: PC = polycarbonate filters, GFF = borosilicate, NC = nitrocellulose filters and SM. Incubation time was 2–3 h, and filter pore was 0.2 μm (except GF/F).

Since the use of inorganic filters is preferable in the ^13^C-PP method, we then examined how ^14^C-POCp and PER results differed between inorganic filters; we also compared these results with those obtained for organic filters. A multi-comparison test (Dunn’s test) showed that SM filters provided ^14^C-POCp and PER statistically similar to those obtained with polycarbonate filters (PC) (*P* = 0.95 and 0.99, respectively). ^14^C-POCp data measured in borosilicate filters (GF/F) were 37–38% significantly higher (*P* < 0.01) than PC and SM values (*P* < 0.01), whereas PER values were 55–57% significantly lower (*P* < 0.01) (Fig. 6). The possible overestimation of POCp and underestimation of PER is likely due to the adsorption of dissolved organic matter in GF/F filters; as previously discussed elsewhere (e.g. Karl *et al.*, 1998). Our results indicate that SM filters can be a good alternative to GF/F filters, commonly used with the ^13^C-PP protocol, particularly if we are interested in determining the dissolved primary production.

Because the incubation time can lead to changes in the phytoplankton community (e.g. exclusion of grazers may promote the accumulation of cells) or favour POC remineralization, we compared ^14^C-POCp and PER measurements obtained for short (2–3 h) incubations periods with those obtained for long incubation (9 h). We found that ^14^C-POCp and PER data obtained from the short incubations were not significantly different from those obtained after 9 h (two sample *t*-test, *t* = 0.15 and 0.16, *df* = 23, and 20, *P* = 0.88 and 0.87, respectively) (data not shown). Thus, we conclude that choosing a 2–3 h incubation period provided sufficient time to obtain significant results.

## DISCUSSION

The phytoplankton productivity regime in the Mediterranean Sea is known to be regulated by the fluctuations between mixing and stratification periods (Estrada, 1996; Pedrós-Alió *et al.*, 1999). In summer, thermal stratification prevents vertical mixing, which restricts the supply of new nutrients into the water column, and primary producers mostly rely on the recycling of nutrients to sustain their metabolic activity and growth (Thingstad and Rassoulzadegan, 1995; Moutin and Raimbault, 2002). The low availability of nutrients in the water column during this season results in low-phytoplankton activity and a relatively constant biomass distribution through the basin (Pedrós-Alió *et al.*, 1999).

In this study, we determined the variability of POCp rates during the summer period, using the ^13^C-PP, and the extensively used ^14^C-PP carbon uptake protocols. Although we did not record changes in temperature (or light intensity) during the incubation, we acknowledge that they could potentially affect our results. However, the photosynthetic rates that were obtained (0.7 mg C m^−3^ h^−1^, on average), lies within the same ranged as those reported by Bosc *et al.* (2004) for the same period. Moreover, our ^13^C-POCp rates also agree with ^13^C-POCp data reported by Marty *et al.* (2008) for their work in the Ligurian Sea, and with the ^14^C-POCp data from Moutin and Raimbault (2002) measured in the Western and Ionian Basins. Carbon assimilation rates per unit Chl*-a* averaged 8 ± 2.3 mg^13^C (Chl*-a*)^−1^ h^−1^, which is similar to the average (7.2 mgC (mg Chl*-a*)^−1^ h^−1^) reported by Marty and Chiavérini (2002) for surface waters at the north-western Mediterranean Sea. The lowest assimilation numbers recorded in this work (<6 mg^13^C (Chl*-a*)^−1^ h^−1^) agree with values (3.5–5.9 mgC (Chl*-a*)^−1^ h^−1^) found in other oligotrophic regions like the subtropical gyres (Karl *et al.*, 1995; Letelier *et al.*, 1996; Marañón *et al.*, 2003).

Although our ^13^C-POCp rates were generally ~0.5 mgC m^−3^ h^−1^, we also identified regions of intense photosynthetic activity between the Ligurian and the Balearic Sea, and in the Algerian Basin. In these regions, frontal structures and cyclonic eddies contribute to the local water column enrichment, promoting the growth of phytoplankton (Lohrenz *et al.*, 1988; Estrada, 1996; Morán *et al.*, 2001). On the other hand, the Tyrrhenian Sea was characterized by the lowest ^13^C-POCp (or ^14^C-POCp) rates in the western basin, confirming that this region is one of the most oligotrophic areas in the Western Mediterranean Sea (Bosc *et al.*, 2004).

We showed that POCp rates measured with the ^13^C-PP protocol were consistent with those obtained with the ^14^C-PP protocol, with the highest degree of similarity for large volumes of samples. Our findings support other studies, showing the major influence of sample size on the primary production results (Sakamoto *et al.*, 1984; Slawyk *et al.*, 1984). Nevertheless, we must also consider that other potential sources of variability can explain discrepancies between the two carbon protocols (Sakamoto *et al.*, 1984; Slawyk *et al.*, 1984, 1988). Mousseau *et al.* (1995) reported that ^13^C-POCp values obtained in the Northwestern Atlantic Ocean were 70% higher than the ones obtained simultaneously with ^14^C. The authors argued that the inconsistencies found between the two methods were not entirely attributed to sample volume, but to changes in incubation temperature and the lack of dark uptake measurements. In our study, we took into account dark uptake rates in both protocols. When we compared ^13^C-POCp rates calculated without any correction for dark uptake with the ^14^C-POCp rates, the data sets remain not significantly different (two sample *t*-test, *t* = 1,22, *df* = 24, *P* = 0.23). Therefore, dark uptake correction seemed to have negligible effects on ^13^C-POCp calculations.

Previously, other authors highlighted the fact that specific filter materials could lead to an overestimation of ^14^C-POCp rates, due to the adsorption of labelled dissolved organic matter (DOC) in the filter (Maske and Garcia-Mendoza, 1994; Karl *et al.*, 1998; Moran *et al.*, 1999). Here, we observed that ^14^C-POCp rates (72-mL samples) measured with organic filters (e.g. GF/F) were significantly higher than those measured with inorganic filters (e.g. PC or silver), and the percentage of exudation (PER) was lower. However, ^13^C-POCp rates gave statistically similar ^14^C-POCp results, in spite of using different filters (i.e. GFF for ^13^C-POCp and PC for ^14^C-POCp). It is likely, as the proportion of POC retained in the filter increases, that the relative importance of any adsorbed material in the filter decreases, compensating the possible overestimation of POC rates measured in GF/F filters when only a small amount of sample is filtered.

The sample size is particularly relevant in oligotrophic systems where there is a high possibility that large and rare cells or phytoplankton colonies (if present) get excluded (Goldman, 1993; Cullen, 2001; Huete-Ortega *et al.*, 2012). The poor representation of these organisms in small samples implies that their contribution to primary production is neglected. Our results showed that ^14^C-POCp rates from small volume samples (72-mL) were 46% lower than those from large samples (1.2-L), for the same protocol ^14^C-POCp but also when using ^13^C-POCp. Other authors previously reported that the underestimation of POCp rates for samples <100 mL could be up to 60% in the tropical and subtropical Atlantic Ocean (Huete-Ortega *et al.*, 2012). Gieskes *et al.* (1979) also found marked discrepancies in ^14^C-POCp rates between samples sizes of 30-mL and 3.8-L samples. The authors suggested that cell damage or a modification of the trophic balance in the smaller samples caused an increase in phytoplankton mortality, hence a reduction of POCp rates and pigment concentration.

Our results demonstrate that the stable^13^C isotope is a reliable alternative to the extensively used radioactive^14^C to determine phytoplankton primary production irrespective of the trophic status of the sea. This finding could provide significant benefits, due to the growing concerns about handling radioactive materials in research vessels. One of the limitations associated with the use of ^13^C is the large volume of water (and space) required to perform the incubations; which in turn restricts the number of samples that can be handled. However, we found that, by processing only a fraction of the sample (~one-third of the GF/F filter), it is possible to obtain results that are similar to those with ^14^C-PP, even in oligotrophic waters. This finding raises the possibility of further improvements to the ^13^C-PP protocol by reducing the incubation volume, thus helping to circumvent problems associated with large-volume incubations.

## CONCLUSIONS

We showed that ^13^C-PP and ^14^C-PP methods produce comparable results when used to measure carbon fixation rates in surface waters of the Mediterranean Sea and that values obtained ranged from 0.5 to 1.5 mgC m^−3^ h^−1^. ^14^C-PP measurements performed simultaneously in <100-mL samples led to lower values (~46% lower than those obtained with the ^13^C-PP method), which we believe can be mostly explained by the difference in samples size. With this study, we provide evidence that the ^13^C-PP method can provide reliable measurements of POCp rates irrespective of the trophic status of the region.
